# Predicting musically induced emotions from physiological inputs: linear and neural network models

**DOI:** 10.3389/fpsyg.2013.00468

**Published:** 2013-08-08

**Authors:** Frank A. Russo, Naresh N. Vempala, Gillian M. Sandstrom

**Affiliations:** ^1^SMART Lab, Department of Psychology, Ryerson UniversityToronto, ON, Canada; ^2^Communication Team, Toronto Rehabilitation InstituteToronto, ON, Canada; ^3^Department of Psychology, University of British ColumbiaVancouver, BC, Canada

**Keywords:** physiological responses, neural networks, music cognition, emotion, computational modeling

## Abstract

Listening to music often leads to physiological responses. Do these physiological responses contain sufficient information to infer emotion induced in the listener? The current study explores this question by attempting to predict judgments of “felt” emotion from physiological responses alone using linear and neural network models. We measured five channels of peripheral physiology from 20 participants—heart rate (HR), respiration, galvanic skin response, and activity in corrugator supercilii and zygomaticus major facial muscles. Using valence and arousal (VA) dimensions, participants rated their felt emotion after listening to each of 12 classical music excerpts. After extracting features from the five channels, we examined their correlation with VA ratings, and then performed multiple linear regression to see if a linear relationship between the physiological responses could account for the ratings. Although linear models predicted a significant amount of variance in arousal ratings, they were unable to do so with valence ratings. We then used a neural network to provide a non-linear account of the ratings. The network was trained on the mean ratings of eight of the 12 excerpts and tested on the remainder. Performance of the neural network confirms that physiological responses alone can be used to predict musically induced emotion. The non-linear model derived from the neural network was more accurate than linear models derived from multiple linear regression, particularly along the valence dimension. A secondary analysis allowed us to quantify the relative contributions of inputs to the non-linear model. The study represents a novel approach to understanding the complex relationship between physiological responses and musically induced emotion.

## Introduction

One of the principal motivations for listening to music is the emotional experience it affords. Although some have argued that this experience does not involve the induction of emotion so much as its perception (Meyer, 1956; Konečni, 2008), few would dispute that physiological change can be evoked while listening to music. Different mechanisms are likely responsible for these physiological changes, ranging from brainstem reflexes to the violation of top-down expectancies defined by culture and personal history (Gabrielsson, 2002; Juslin and Västfjäll, 2008). These physiological changes can be assessed non-invasively through continuous measurement of heart rate (HR), respiration, skin conductivity, facial muscle activity, and other peripheral measures. Because different types of felt emotion have been associated with different patterns of physiological change (Krumhansl, 1997; Nyklicek et al., 1997; Rainville et al., 2006; Lundqvist et al., 2009), it is reasonable to investigate the extent to which physiological responses to music can be used in and of themselves to predict felt emotion.

Both discrete and dimensional models of emotion have been used to conceptualize emotional responses to music^1^. Discrete models (e.g., Ekman, 1992, 1999) have the advantage of avoiding assumptions about the manner in which emotions may be related to one another, thus allowing for representation of mixed emotions (e.g., bitter-sweet as a combination of happiness and sadness). Dimensional models (e.g., Hevner, 1935, 1936; Russell, 1980) characterize emotions with respect to an n-dimensional space, thus enabling quantification of the psychological distance between any two emotions as well as characterization of the relationship between a set of emotions (e.g., bored has been conceptualized as a combination of sadness and fatigue).

Research on music and emotion over the last decade has tended to prefer dimensional models. In an effective demonstration of this approach, Bigand et al. (2005) identified a collection of music representing points across the entire surface of the two-dimensional grid constituted by the intersection of valence and arousal (henceforth valence-arousal grid; Russell, 1980; Schubert, 2004). Valence refers to the hedonic dimension of emotion, ranging from pleasant to unpleasant. Physiological correlates of the valence dimension in musically induced emotion include zygomaticus major and corrugator supercilii activity (e.g., Witvliet and Vrana, 2007; Lundqvist et al., 2009). Arousal refers to the mobilization of energy, ranging from calm to excited. Physiological correlates of the arousal dimension include autonomic measures such as HR, respiration, and galvanic skin response (e.g., Iwanaga et al., 1996; Krumhansl, 1997; Baumgartner et al., 2005; Etzel et al., 2006; Sandstrom and Russo, 2010). Three-dimensional models of emotion have also been applied to music (Illie and Thompson, 2006) but the advantages of including a third dimension (e.g., tension-arousal) are unclear at this stage of research (Eerola and Vuoskoski, 2011).

Past research on musically induced emotion and physiological responses has almost exclusively been limited to linear models. In contrast, similar research on subjective feelings in other contexts (e.g., video games), have begun to use non-linear computational models (Mandryk and Atkins, 2007; Fairclough, 2009). Non-linear computational models such as those generated by artificial neural networks have great potential for adding to the understanding of music and emotion as they allow for prediction of felt emotion without the artificiality that is introduced by requiring a listener to consciously reflect on their emotional experience. However, there are only a few studies of musically induced emotions that anticipate the computational approach taken here.

In a pioneering study, Kim and André (2008) trained an automatic musical emotion recognition system based on physiological data that was collected from three listeners. Their measures included HR, respiration, skin conductance (SCL), and electromyography of the trapezius muscle. They used an extended linear discriminant analysis to classify the emotion that listeners experienced as falling into one of the four quadrants of the valence-arousal grid. Although the model achieved a reasonable level of recognition accuracy (70%), the small number of listeners that were used to train the model greatly limits its generalizability. In addition, because the model was designed to classify a musical excerpt into one of four categories (the quadrants of the valence-arousal grid), it was unable to predict subtle differences within the same quadrant or to account for variation along a particular dimension of emotion (e.g., valence).

Coutinho and Cangelosi (2009) used a neural network approach to predict continuous variation along the valence and arousal dimensions of musically induced emotion. The continuity of measurement represents an important departure from Kim and André (2008). Input to the neural network model involved combinations of low-level psychoacoustic features (timbre, mean pitch, pitch variation, and dynamics). The model was trained on three excerpts and tested on an additional three excerpts. The model was effective in predicting moment-to-moment changes in felt emotion.

In a subsequent study by Coutinho and Cangelosi (2011), psychoacoustic as well as physiological features were incorporated into neural network models for predicting musically induced emotions. Psychoacoustic features included loudness, pitch level, pitch contour, tempo, texture, and sharpness. Physiological features included HR and SCL. Results showed that the physiological features were able to provide only a slight increase in explained variance beyond that accounted for by the psychoacoustic features alone. The addition of other physiological features such as those considered here may have helped to further increase explanatory power. However, as acknowledged by the authors, the models derived from psychoacoustic features were already quite powerful and the variable lag in different channels of physiological response complicates continuous prediction.

In the current study, five channels of physiological data were obtained while participants listened to music excerpts selected to represent each quadrant of the valence-arousal grid: high arousal, positive valence (*Happy)*, high arousal, negative valence (*Agitated)*, low arousal negative valence (*Sad)*, and low arousal, positive valence (*Peaceful)*. All excerpts were drawn from the classical era so as to minimize variability in responses due to genre. Listeners provided global ratings of felt emotion (taking into account the entire excerpt). Linear regression and neural network models were developed using only physiological features as input and subjective appraisals of felt emotion as output. One promise of this particular approach that emphasizes physiological inputs is that it may inform the development of future models that are capable of predicting the appraisals of a particular listener, or a particular type of listener listening to a particular genre of music.

## Methods

### Participants

We recruited 32 undergraduate students through our departmental participant pool. Twelve of the participants had some proportion of missing physiological data in one or more of the channels due to measurement error. The most common error was that our recordings of facial muscle activity were interrupted for a portion of the trial due to electrodes losing surface contact (mainly due to an accumulation of perspiration toward the end of the session). Our analyses only considered data from those 20 participants providing a complete data set (17 females, 1 male, 2 undeclared). On average these participants were 25 years of age (*SD* = 9.2) with 1.7 years of individual music training (*SD* = 2.9) and 2 years of group training (*SD* = 2.8).

### Stimuli and apparatus

Our stimuli consisted of 12 classical music excerpts (M1–M12) from 12 different composers, as shown in Table 1. Three excerpts were chosen to represent each of the four emotion quadrants of the valence-arousal grid: high arousal, positive valence (*Happy)*, high arousal, negative valence (*Agitated)*, low arousal negative valence (*Sad)*, and low arousal, positive valence (*Peaceful)*. We used an excerpt of white noise, equated with the root-mean-square (RMS) level collapsed across the music tracks, as our baseline stimulus. A unique baseline was computed for each participant and trial. RMS-matched white noise provides a situational context that should be comparable to that of the music excerpts while remaining emotionally neutral, thus allowing us to isolate effects on physiology due to emotion (Nyklicek et al., 1997; Sokhadze, 2007). These excerpts were chosen based on previous work investigating emotional responses to music (Nyklicek et al., 1997; Bigand et al., 2005). All excerpts were 40 s in duration, normalized to a set RMS value, and presented at ~75 dB SPL over Sennheiser HD 580 Precision headphones.

**Table 1:** **Twelve music excerpts with composers, emotion quadrants, and mean valence/arousal ratings**.

**Excerpt**	**Composer**	**Composition**	**Quadrant**	**Mean valence**	**Quadrant valence**	**Mean arousal**	**Quadrant arousal**
M1	Bartok	Sonata for 2 pianos and percussion (Assai lento)	Agitated	5	4.1	6.35	6.98
M2	Shostakovich	Symphony No. 8 (Adagio)	Agitated	3.35		7.45	
M3	Stravinsky	Dame sacrale (Le Sacre du Printemps)	Agitated	3.95		7.15	
M4	Beethoven	Symphony No. 7 (Vivace)	Happy	6.6	6.38	6.35	6.7
M5	Liszt	Les Preludes	Happy	5.75		6.25	
M6	Strauss	Unter Donner und Blitz	Happy	6.8		7.5	
M7	Bizet	Intermezzo (Carmen Suite)	Peaceful	6.6	6.1	2.85	2.77
M8	Dvorak	Symphony No. 9 (Largo)	Peaceful	5.95		2.65	
M9	Schumann	Traumerei	Peaceful	5.75		2.8	
M10	Chopin	Funeral March, Op. 72 No. 2	Sad	4.85	4.4	2.55	3.48
M11	Grieg	Aase's death (Peer Gynt)	Sad	4.05		4.15	
M12	Mozart	Requiem (Lacrimosa)	Sad	4.3		3.75	

Participants were tested in a double-walled sound attenuation chamber (Industrial Acoustics Company). Five simultaneous channels of physiological data were sampled at 1000 Hz using a Biopac MP100 data acquisition system (Biopac Systems, Santa Barbara, CA) under the control of a Mac mini computer running AcqKnowledge software (Biopac Systems), version 3.9.2 for Mac: Measurement details for each channel are provided below.

#### Skin conductance (SCL)

Isotonic conductant gel was applied to two TSD203 Ag-Agcl electrodes. The electrodes were attached to the distal phalanges of the index and ring fingers of the non-dominant hand using Velcro straps, and connected to the GSR100C amplifier to measure SCL.

#### Heart rate (HR)

One TSD200 photoplethysmogram transducer was attached by a Velcro strap to the distal phalange of the middle finger of the non-dominant hand. This transducer was connected to the PPG100C amplifier to measure capillary expansion through an infrared sensor, and thus indirectly measure the HR.

#### Respiration rate (Resp)

One TSD201 respiration belt was comfortably tightened around the upper part of the abdomen and attached to the RSP100C amplifier to record changes in thoracic or abdominal circumference.

#### Facial muscle activity (Zyg and Corr)

Shielded 4 mm silver-silver chloride (Ag/AgCI) miniature surface electrodes (Biopac, EL 208 S) were filled with electrode gel. Two of the electrodes were placed on the zygomaticus major and two on corrugator supercilii muscle regions, both on the left of the face separated by a distance of 25 mm and attached over the ear to the EMG100C amplifier to measure muscle activity.

### Procedure

Participants heard all 12 music excerpts in one session. Each music excerpt was preceded by 30 s of white noise, and followed by 50 s of silence. The 12 music excerpts were arranged in four different random orders. Each participant was randomly assigned to one of the four orders.

Immediately after hearing each music excerpt, participants reported the valence and arousal of the felt emotion using the Self-Assessment Manikin (Bradley and Lang, 1994). This procedure incorporates pictures to clarify Likert-type ratings from 1 to 9 on valence (least pleasant/most pleasant) and arousal dimensions (least excited/most excited). In addition to valence and arousal, participants provided a score on a scale from 1 to 4 regarding their familiarity with the excerpt, where 1 corresponds to “I've never heard this song before,” 2 corresponds to “I think I might have heard this song once or twice before,” 3 corresponds to “I am somewhat familiar with this song,” and 4 corresponds to “I am very familiar with this song.” The mean familiarity ratings were generally quite low; all excerpts had a mean familiarity rating lower than 2.5, and the mean excerpt familiarity rating was 1.78 (*SD* = 0.30).

### Data preparation and preliminary analyses

In order to test for effects of presentation order and music training (number of years), a preliminary analysis of covariance was run on each dimension of felt emotion. For each analysis, the within-subjects factor was music excerpt and the between-subjects factor was presentation order; music training was entered as the covariate. These analyses confirmed that the effects of presentation order, and music training, were non-significant, *F*'s < 1, while the effects of music excerpt were significant, *F*'s_(11, 165)_ = 6.03 and 22.86, *p* < 0.001.

**Figure 1:**
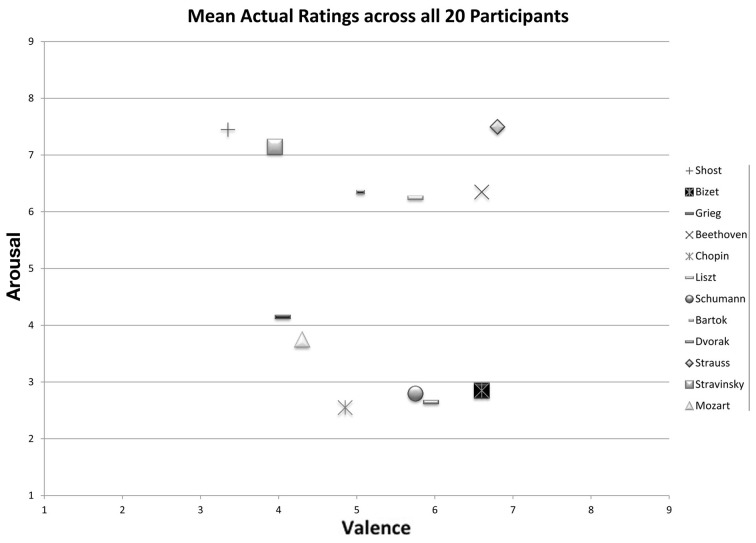
**Mean participant valence/arousal ratings (on a scale of 1–9) for all 12 excerpts (training and test sets combined)**.

As seen in Figure 1 and reported in Table 1, the mean valence and arousal ratings for excerpts were well-distributed across the valence-arousal grid, and they aligned in the expected manner according to the four quadrants (happy, agitated, sad, peaceful). The mean valence ratings ranged from 3.35 (*SD* = 1.84) for M2 (Shostakovitch) to 6.8 (*SD* = 1.94) for M6 (Strauss). The mean arousal ratings ranged from 2.55 (*SD* = 1.54) for M10 (Chopin) to 7.5 (*SD* = 1.19) for M6 (Strauss). The inter-subject variability was comparable between valence and arousal ratings (Mean *SD* = 1.82 and 1.80, respectively).

Signal processing of physiological data involved the application of high-pass (HP) and/or low-pass (LP) filters, and where applicable, rate conversion using a peak detection algorithm with minima and maxima: SCL (no filters), Resp (no filters; Min/Max = 5/180), HR (*LP* = 3 Hz; *HP* = 0.5 Hz; Min/Max = 40/180), EMG (*HP* = 1 Hz; *LP* = 500 Hz). The data from each channel was standardized independently for each participant (converted to z-scores). A single feature value was then determined for each excerpt by subtracting mean values obtained in the final 20 s of white noise (baseline) from the mean of standardized values obtained in 30 s of each trial (i.e., the first 10 s of baseline and music were excluded to avoid capturing a startle effect). Filtering, standardization, and baseline subtraction was completed in FeatureFinder (Andrews et al., 2011), a freely available Matlab toolbox for custom analysis of physiological signals.

### Linear correlation and multiple linear regression

As a first step toward capturing the patterns by which these five physiological features accounted for valence and arousal ratings, we checked to see if there was a correlation between the physiological features and the mean valence-arousal (VA) ratings for the 12 music excerpts. Valence ratings were not significantly correlated with any of the physiological variables, *p*'s > 0.1. Arousal ratings were correlated significantly with HR, *r*_(10)_ = 0.88, *p* < 0.001, and marginally with Resp, *r*_(10)_ = 0.53, *p* = 0.08, but not with Zyg, Corr, or SCL, *p*'s > 0.1.

As a next step, we performed multiple linear regression with stepwise forward entry to determine whether there was a linear relationship between some combination of the physiological features and the VA ratings. The caveat here is that the models need to be interpreted with caution given that the ratio of sample size (number of excerpts) to predictors (physiological features) is smaller than accepted norms (Harrell, 2001). For valence, no significant model emerged. The best linear regression model for arousal included HR (*p* < 0.01) and Resp (*p* = 0.07), accounting for 85.2% of the variance, *F*_(2, 11)_ = 25.8, *p* < 0.001. These results suggest that while a linear combination of the physiological features may account for arousal, no linear combination adequately accounts for valence.

### Artificial neural networks

One way of exploring non-linear combinations of physiological features is through the use of artificial neural networks. Although artificial neural networks have been applied extensively for classification and detection tasks in domains such as object and speech recognition, they have been relatively underutilized in music cognition (see however, Bharucha, 1987; Stevens and Latimer, 1992; Krumhansl et al., 2000; Vempala and Maida, 2011). In the current study, we applied neural networks as a non-linear regression function to predict valence and arousal ratings using physiological features as inputs. Our implementation was a supervised feedforward neural network with backpropagation, also known as a multilayer perceptron (Rumelhart et al., 1986; Bishop, 1996; Haykin, 2008).

**Figure 2:**
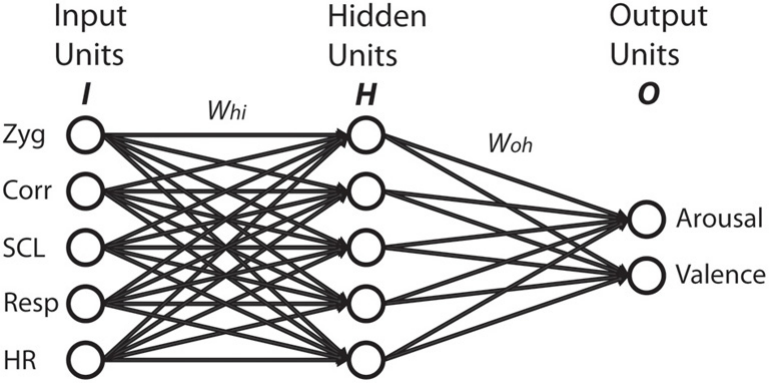
**Neural network with five input units, five hidden units, and two output units.** *W*_*hi*_ indicates connection weights from input units to hidden units. *W*_*oh*_ indicates connection weights from hidden units to output units.

First, we defined the inputs and outputs for the network. From the 12 music excerpts, we arbitrarily chose two out of three from each quadrant for our training set: M1/M2 for *agitated*; M4/M5 for *happy*; M7/M8 for *peaceful*; and M10/M11 for *sad*. The test set consisted of the remaining four excerpts: M3 for *agitated*; M6 for *happy*; M9 for *peaceful*; and M12 for *sad*. The network's task was to predict the valence and arousal ratings based on the five physiological features. The training set consisted of eight input and output vectors. Each input vector had five values, one for each physiological feature, collapsed across participants. The corresponding output vector had arousal and valence values, again collapsed across participants. To maximize network learning (within and across channels), all of the physiological inputs were scaled to a value between 0 and 1 (Bishop, 1996). To avoid overfitting the network, we kept the number of hidden units equal to the number of input units. Thus, the network architecture consisted of five input units (one for each physiological feature), a single hidden layer with five units, and two output units as shown in Figure 2.

Next, we implemented the network in Matlab. The following procedure was used to train the network.

1. Connection weights *W*_*hi*_ (input units to hidden units) and *W*_*oh*_ (hidden units to output units) were initialized to random numbers between 0 and 0.05. Input vectors were fed to the network from the training set in a randomized order. Inputs were passed through a sigmoidal function, multiplied with the connection weights *W*_*hi*_, and summed at each hidden unit.
2. Hidden unit values were obtained by passing the summed value at each hidden unit through a sigmoidal function. These values were multiplied with the connection weights *W*_*oh*_, summed at each output unit, and passed through a sigmoidal function to arrive at the final output value.
3. Network outputs were compared to mean valence and arousal ratings and the error was computed. The backpropagation algorithm was applied and changes in connection weights were stored. At the end of the entire epoch, connection weights were updated with the sum of all stored weight changes.
4. The network was trained for 80,000 epochs by repeating step 2 to reduce the mean squared error to less than 0.02. During training, the learning rate parameter was set to 0.1.

We repeated this training procedure for 20 trials (i.e., 20 instances of fully trained networks). For each trial we re-initialized the network connection weights, repeated the training procedure on the same set of eight excerpts and tested the network on the remaining four.

**Figure 3:**
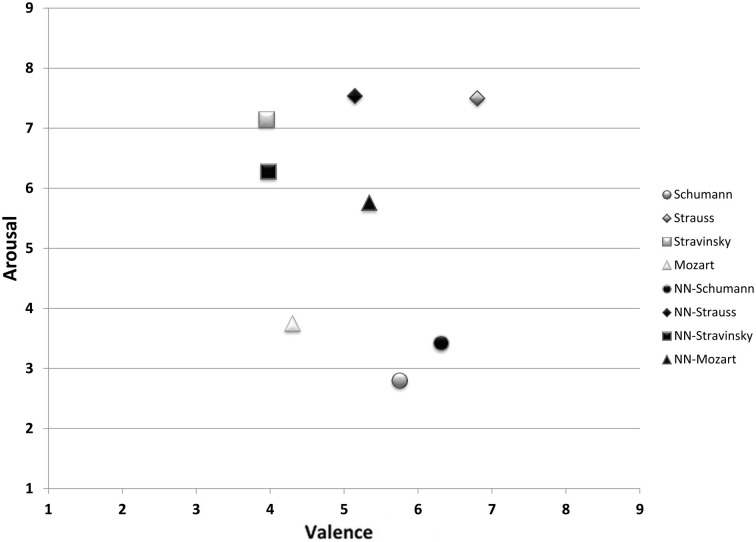
**Mean participant valence/arousal ratings (on a scale of 1–9) for the test set and corresponding mean neural network outputs.** *NN* indicates neural network output.

Figure 3 reports the average network performance for the four test excerpts in comparison with participant ratings. The network performed particularly well for M3 (Stravinsky) and M9 (Schumann). Predicted values for M6 (Strauss) were very close to the expected value on the arousal dimension and 1.6 scale units off on the valence dimension. M12 (Mozart) yielded the worst overall network performance, with an error of 1 scale unit on valence and 2 scale units on arousal.

To quantify the network's performance, we calculated the Euclidean distance between mean network-predicted outputs and mean participant ratings for valence and arousal. Table 2 shows the network's performance for each selection and average performance across all four selections for valence and arousal. The network's mean performance error for valence was 0.82 (on a scale from 1 to 9), indicating that the network accuracy for valence was 89.75%. The network accuracy for arousal was 88.92%.

**Table 2:** **Performance of neural network and linear regression performance for each of the 4 test excerpts**.

**Excerpt**	**Neural network**		**Multiple regression**	
	**Valence error**	**Arousal error**	**Valence error**	**Arousal error**
M3 (Stravinsky; agitated)	0.025	0.868	3.795	0.59
M6 (Strauss; happy)	1.656	0.04	9.363	0.407
M9 (Schumann; peaceful)	0.56	0.626	8.155	5.459
M12 (Mozart; sad)	1.039	2.01	8.05	3.989
Mean error	0.82	0.886	7.341	2.611
Mean error %	10.25	11.08	91.76	32.64
Mean performance accuracy %	89.75	88.92	8.24	67.36

Having quantified the network's performance, we sought to determine whether the neural network approach yielded an improvement in emotion prediction over multiple linear regression. In order to derive comparable models, we computed regression models using stepwise forward entry based on data from the eight test excerpts (note that the regression models reported above had used all 12 excerpts). Given the small number of cases, it is not surprising that a significant model did not emerge. Nonetheless, to allow performance comparisons we computed the Euclidean distance between predicted outputs of the revised regression models and the mean participant ratings. As observed in Table 2, performance was extremely poor for the linear model of valence, with accuracy of 8.24%. Performance was somewhat better for the linear model of arousal, with accuracy of 67.36%. Table 3 tells a similar story about the relative performance of the two approaches but from the perspective of RMSE and correlation between model outputs and mean valence/arousal ratings (*df* = 10). Collectively, these performance results confirm that a linear model of the five physiological features is inferior to a non-linear model derived by an artificial neural network, particularly for the valence dimension.

**Table 3:** **Two measures of performance for neural network and linear regression based on all 12 excerpts: (1) RMSE and (2) correlation with mean valence/arousal ratings**.

	**Neural network**		**Linear regression**	
	**Valence**	**Arousal**	**Valence**	**Arousal**
RMSE	0.09	0.11	0.1	0.17
Correlation *(r)*	0.79	0.91	0.73	0.77
Explained variance (*r*^2^)	62.4%	82.8%	53.3%	59.3%

Our next goal was to understand the importance of each physiological feature in terms of its contribution to the non-linear solution. To determine the relative contributions of each feature, we used a method derived by Milne (1995) that was designed for neural networks like ours with a single hidden layer. Milne's method is an improvement over a method first proposed by Garson (1991) that does not determine relative size of contributions in networks that include a combination of positive and negative connection weights. Another method proposed by Wong et al. (1995) allows a determination of relative size but the sign of the contribution is lost. In contrast, Milne's method allows for the determination of relative size and direction of each contribution.

**Figure 4:**
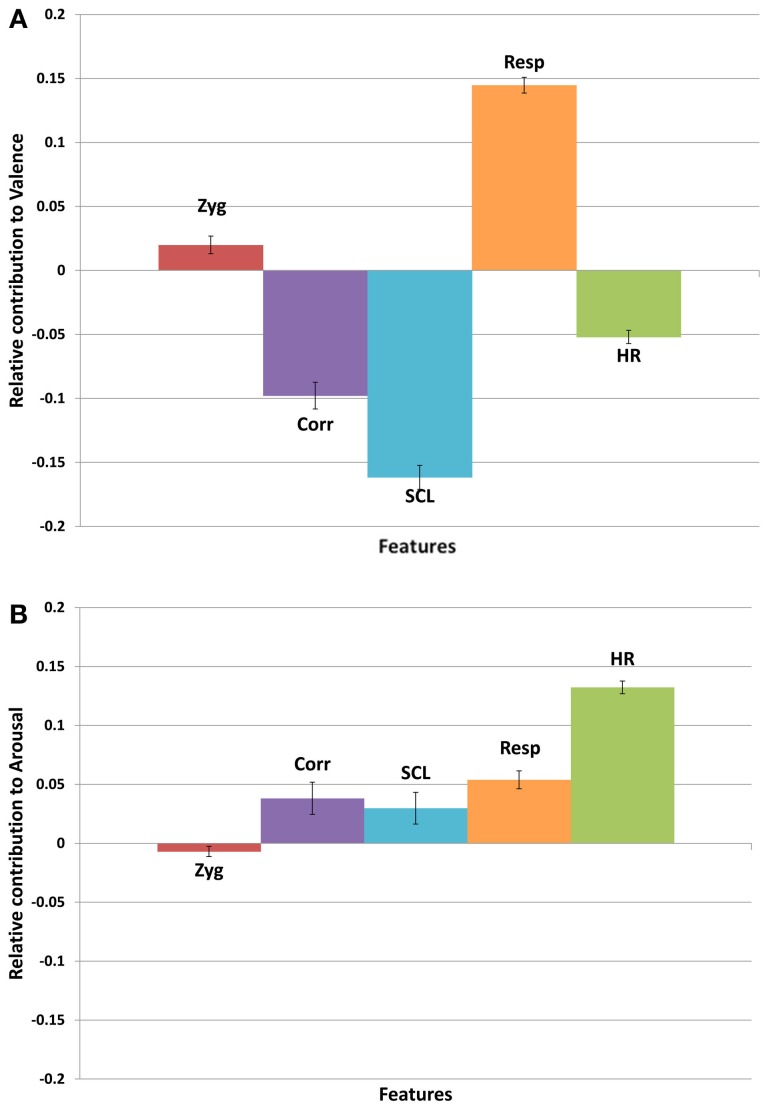
**Contribution of each physiological feature toward judgments of (A) valence, and (B) arousal, based on weight proportion.** A negative value indicates that the physiological feature is higher for negative valence, or low arousal, respectively.

Using Milne's method, we determined the relative size and direction of each feature's contribution for each of the 20 trials (see Figure 4). The relative size data were then subjected to separate analyses of variance for valence and arousal with physiological feature as the repeated measure. There was a significant effect of physiological feature on relative size of contributions for valence, *F*_(4, 76)_ = 198.7, *p* < 0.001, and for arousal, *F*_(4, 76)_ = 23.1, *p* < 0.001^2^.

## Discussion

The neural network models that we developed on the basis of eight training excerpts were highly accurate in their prediction of valence and arousal ratings for four test excerpts (89.75 and 88.92%, respectively). The predictive power of these non-linear computational models was better than linear models that we implemented, particularly for the valence dimension. On the basis of the current study, it seems that valence cannot be adequately predicted using linear regression of features derived from physiology measures alone, but valence can be predicted using non-linear functions such as those found in neural networks. Although the network architecture prevents us from fully dissecting its non-linear function, we were able to assess the relative size and the direction of each physiological feature's contribution.

In order to test the relative size of contributions, we established a threshold corresponding to expected performance given the null hypothesis (i.e., equal contributions from each physiological feature; henceforth the null threshold). For valence, the relative size of contributions of SCL, Resp, and Cor were above the null threshold. Consistent with findings from prior studies, valence was negatively related to SCL and Cor (Krumhansl, 1997; Baumgartner et al., 2005) and positively related to Resp (Etzel et al., 2006; Witvliet and Vrana, 2007). For arousal, the relative size of contributions of HR and Resp were above the null threshold; the direction of both contributions was positive and thus consistent with prior research (Iwanaga et al., 1996; Krumhansl, 1997; Etzel et al., 2006; Sandstrom and Russo, 2010) as well as the results of our linear regression. Although the null threshold described above is somewhat arbitrary, we consider it non-trivial that the directionality of super-threshold contributions to felt emotion revealed in the neural networks is anticipated by prior research that employed linear modeling methods.

Our results suggest that estimates of felt emotion can be derived from neural network models that take input solely from peripheral physiological measures. While this might be considered a satisfactory outcome from a computational standpoint, it is important to ask what impact this might have for emotion science. In our view, the potential impact is greatest in the development of theory that seeks to explain the emotional trajectory of longer excerpts of music. If we have a fully validated model that does a good job of predicting subjective appraisals for an individual or a particular type of listener, then we avoid the problem of artificiality that is introduced by requiring the listener to consciously reflect on their emotional experience. Instead, we can ask the listener to experience the music as they would outside of the context of a laboratory, using the model to provide the output that the subjective appraisals are intended to provide. The output could then be explicated on the basis of acoustical, psycho-acoustical or musical factors abstracted from the music.

There are several limitations to acknowledge in this study. First, the neural network model was trained on the basis of only eight music excerpts. Although these excerpts were selected so as to span the entire valence-arousal grid, the small number of excerpts greatly limits generalizability of the findings even for excerpts of the same genre. Related to this first point is the potential problem of overfitting that may have occurred because there are more connections than training excerpts. It is quite possible that our neural network would be less accurate in the face of new excerpts from the same genre that differ in their emotional tenor. The small number of excerpts also prevents us from making statistical inference on the predictive power of linear and neural network models. Second, our excerpts were homogeneous with regard to genre of music—they were all instrumental classical music, albeit from different stylistic traditions (e.g., Bartok vs. Beethoven). Third, the inputs and outputs to the model were derived from a group of listeners that were treated as members of a homogeneous population. The inputs and outputs to the neural network models were based on aggregate data (collapsing across participants). We assume that a new randomly selected sample would yield similar aggregate data. However, participants varied with respect to felt emotion and their physiological responses. In all likelihood, this variability was influenced by their music preferences (Rentfrow and Gosling, 2003; Salimpoor et al., 2009) and the extent to which they are absorbed by music (Sandstrom and Russo, 2013). Future work should test the neural network model trained in the current study on an independent group of participants. In addition, it will be important to develop new models on larger participant samples and larger collections of music. One direction will be to train a domain-general model that is capable of performing well with any type of listener or genre of music. Another, potentially more important, direction will be to train domain-specific models that are tailored to particular types of listeners and genres. The former should be robust across contexts but mediocre in its predictive power. The latter will have increased power so long as it is tested in contexts that are consistent with training.

Another important limitation of this study is that there was no representation of time in the models (c.f., Coutinho and Cangelosi, 2011). The experience of emotion in music often follows a trajectory (e.g., tension-release), in which the emotional response to a section of music will depend in part on the emotional state of the observer in the preceding section (Dibben, 2004). One means of incorporating time is through a simple recurrent network (e.g., an Elman network), which uses context from the previous time-step as additional input for the current time-step (Elman, 1993). Although previous studies have used Elman networks to predict variability in subjective reports of felt arousal and valence (Coutinho and Cangelosi, 2009, 2011; Vempala and Russo, 2012), physiological features have only led to limited explanatory gains over psychoacoustic features. One reason for this shortcoming may be the variable timecourse of physiological features (e.g., facial responses tend to be faster than changes in galvanic skin response). One potential solution that sidesteps the problem is to use time-steps that are long enough to accommodate variability in the time course of physiological features (e.g., no shorter than 5 s).

A final limitation of this study is that we have no way of determining whether the predictive features derived from physiological measures were the cause or effect of subjective appraisals. While we have treated the features as inputs and the appraisals as outputs, we are not suggesting that the physiological responses necessarily give rise to the appraisals. It is also possible that the relations are bidirectional in some manner, contributing collectively to the overall experience of emotion (Gross and Barrett, 2011).

## Conclusion

Our results demonstrate that computational methods may be used to predict musically induced emotion on the basis of physiological features alone. Neural networks led to stronger predictions than linear modeling approaches, particularly along the valence dimension. The results of this study contribute to our understanding of the powerful emotional experience that leads so many people to listen to music.

### Conflict of interest statement

The authors declare that the research was conducted in the absence of any commercial or financial relationships that could be construed as a potential conflict of interest.

## Footnotes

^1^One further class of models that has elements of both discrete and dimensional approaches is the domain-specific model developed specifically for music by Zentner et al. (2008).

^2^In order to account for the fact that the networks from the 20 trials were not independent (although the weights were randomized prior to training, the outputs and type of neural network were the same on every trial), we used hierarchical linear modeling, using the HLM for Windows software (Version 6.08; Raudenbush et al., 2004). HLM allows the slopes of the relationship between channel and proportion contribution to be different for each network trial. We created four dummy codes at level 1 (within-network trial) to represent the different channels, using Zyg as the reference group. There were no level 2 (between-network trial) variables. Analyses revealed that the proportion contributions of Corr, SCL, Resp, and HR were all greater than the proportion contribution of Zyg for both valence (*p*'s < 0.03) and arousal (*p*'s < 0.02). These results confirm that channels differ in their proportion contributions. The variance component for the intercept was not significant for either valence or arousal (*p*'s < 0.5), indicating that the proportion contribution intercepts did not vary significantly across network trials.
